# Unprecedented variation pattern of plastid genomes and the potential role in adaptive evolution in Poales

**DOI:** 10.1186/s12915-024-01890-5

**Published:** 2024-04-29

**Authors:** Hong Wu, De-Zhu Li, Peng-Fei Ma

**Affiliations:** 1 Germplasm Bank of Wild Species and Yunnan Key Laboratory of Crop Wild Relatives Omics, Kunming Institute of Botany, Chinese Academy of Sciences, Kunming, 650201 Yunnan China; 2 University of Chinese Academy of Sciences, Beijing, 100049 China

**Keywords:** Poales, Plastomic variation, Heterogeneous substitution rate, Repeat, Inversion, Adaptive evolution

## Abstract

**Background:**

The plastid is the photosynthetic organelle in plant cell, and the plastid genomes (plastomes) are generally conserved in evolution. As one of the most economically and ecologically important order of angiosperms, Poales was previously documented to exhibit great plastomic variation as an order of photoautotrophic plants.

**Results:**

We acquired 93 plastomes, representing all the 16 families and 5 major clades of Poales to reveal the extent of their variation and evolutionary pattern. Extensive variation including the largest one in monocots with 225,293 bp in size, heterogeneous GC content, and a wide variety of gene duplication and loss were revealed. Moreover, rare occurrences of three inverted repeat (IR) copies in angiosperms and one IR loss were observed, accompanied by short IR (sIR) and small direct repeat (DR). Widespread structural heteroplasmy, diversified inversions, and unusual genomic rearrangements all appeared in Poales, occasionally within a single species. Extensive repeats in the plastomes were found to be positively correlated with the observed inversions and rearrangements. The variation all showed a “small-large-moderate” trend along the evolution of Poales, as well as for the sequence substitution rate. Finally, we found some positively selected genes, mainly in C_4_ lineages, while the closely related lineages of those experiencing gene loss tended to have undergone more relaxed purifying selection.

**Conclusions:**

The variation of plastomes in Poales may be related to its successful diversification into diverse habitats and multiple photosynthetic pathway transitions. Our order-scale analyses revealed unusual evolutionary scenarios for plastomes in the photoautotrophic order of Poales and provided new insights into the plastome evolution in angiosperms as a whole.

**Supplementary Information:**

The online version contains supplementary material available at 10.1186/s12915-024-01890-5.

## Background

The plastid is the metabolically active semi-autonomous organelle in plants, which is mainly involved in photosynthesis and can also participate in many biosynthesis [1]. The plastid, together with the nucleus and mitochondrion, are the three genetic compartments in the plant cell. The plastid genome (plastome) is generally circular in structure, with a typical size ranging from 120 to 160 kb in flowering plants [2]. In addition, the plastome in most plants have a tetrad structure with two equal-sized inverted repeats (IRa and IRb, ~ 20–28 kb), dividing the whole genome into a large single-copy region (LSC, ~ 80–90 kb) and a small single-copy region (SSC, ~ 16–27 kb) [3]. Generally, a total of 110–120 genes including protein-coding, ribosomal DNA (rDNA), and transfer RNA (tRNA) genes are encoded in the plastome with and the majority of them functioning photosynthesis [4].

The plastomes have long been documented to be conserved in evolution and have a moderate molecular sequence evolution rate as compared to the nucleus and mitochondrial genomes in plants [5]. However, with the increasing body of sequenced genomes, a certain degree of variation was observed for plastomes [6–13]. This is somewhat expected as the heterotrophic plants usually have lost the ability of photosynthesis, accompanied by the loss of photosynthesis-related genes and degradation of plastome with many genomic rearrangements, such as in *Petrosavia* [14] and *Cuscuta* [12, 13, 15]. Furthermore, plastomic variation can also be found in certain photosynthetic lineages, e.g., lycophyte, Fabaceae, subfamily Lobelioideae in Campanulaceae and *Pelargonium* [16–21]. The documented variations mainly involve expansion/contraction or even loss of IR, gene duplication, gene and intron loss, inversion, and genomic rearrangement [14, 20, 22–27].

Within the plastome, the IR region is more conserved in size and gene content, as well as lower substitution rate, compared to the single-copy regions [28]. The ribosomal genes (*rrn16*, *23*, *4.5*, and *5*), together with seven protein-coding genes (*rpl2*, *23*, *ndhB*, *rps7*, *12*, *ycf2*, and part of *ycf1*) and several tRNA genes, are usually located in the IR region [2, 19]. It is speculated that IR can stabilize the plastome through a repair mechanism induced by homologous recombination [29, 30], possibly contributing to the slow evolution of plastome [2, 28]. However, the expansion and contraction of the IR region has frequently been observed such as expansion in *Pelargonium* and *Petroselinum* [19, 31] and contraction in *Erodium*, *Trifolium*, and *Pinus* [28], being able to cause extensive variation in plastome size. Even the complete loss of IR was found in angiosperm families of Fabaceae, Cactaceae, Arecaceae, and Putranjivaceae [2, 23, 24, 27, 32]. The loss of IR could be also found in the coniferous plants with short inverted repeat (sIR) generated, ranging in size from tens to more than 1000 base pairs, and the sIR has the ability to mediate different isomers of plastomes [33–35]. The IR could even be transformed into direct repeat (DR) by multiple events of inversions such as in *Selaginella* [21]. Furthermore, three IR copies was found in *Chamaetrichon* [36]. In addition to IR, the repeats larger than 1 kb can also provide homologous sequences for the recombination-dependent replication (RDR) [37], resulting in the coexistence of different alleles in an individual, called as plastome heteroplasmy [38].

In addition to the genomic rearrangements associated with the IR region, the occurrence of gene duplication, gene and intron loss, and inversions has also been reported in the plastome [24]. Gene duplication is generally rare and mainly due to the IR expansion, such as those found in the plastomes of *Eleocharis*, *Arbutus unedo*, and *Asarum* [39–41]. On the other hand, gene and intron loss are more frequently observed and approximately 62 independent loss events occurred across the evolution of flowering plants [24]. Multiple losses are observed for *ndhA*-*K*, *infA*, and *rps16* and for introns in the *clpP*, *rpl2*, *rpl16*, *rpoC1*, and *rps12* [42–44]. The occurrence of inversions has been reported in multiple lineages (e.g., Fabaceae, Poaceae, Passifloraceae, *Pelargonium*, *Scaevola*, *Trachelium*, *Jasminum*, and *Oenothera*) [23, 24] and sometimes could be used as a phylogenetic marker. Its occurrence could be caused by the presence of sequence repeats at both ends of it [45, 46] and those larger than 1 kb can lead to the rearrangement of plastome [16, 25]. The changes of gene order by inversions have been suggested to play a role in the adaptative evolution of green plants and algae [47]. Accelerating sequence evolution was also detected in the highly rearranged plastomes of Geraniaceae [27] and previous studies suggested a significantly faster evolutionary rate of plastomic DNA in Poales compared to the other commelinid groups [48, 49]. Moreover, the evolutionary rate may be heterogeneous during the evolution of Poales with low in early-diverging group while high rate in Poaceae [11, 50–52].

As described above, amounts of studies revealed the genomic variation of plastid in plants from different aspects in light of an overall conserved evolutionary history. However, the evolutionary pattern of plastomes in angiosperms, especially in order at the family level, remains scarce. As one of the largest and the most economically important order of plants [53], Poales was found to show a certain degree of plastomic variation in previous studies [39, 54–59], despite being photoautotrophic plants. For example, three inversions (~ 28 kb, ~ 6 kb, and < 1 kb in size) and multiple gene and intron losses have long been documented in the plastomes of grasses (Poaceae) [55]. Furthermore, similar inversions also occurred in the plastomes of Ecdeiocoleaceae and Joinvilleaceae as closely related families of Poaceae, as well as gene loss such as *accD* [56, 57]. More variation was revealed in the Cyperaceae and two sequenced *Eleocharis* species had distinct characteristics including larger plastome size of about 200 kb, high rate of sequence recombination, low GC content and gene density, and a large amount of repetitive DNA sequence with at least four different plastomic configurations existing in each species [39]. Recent sequencing of 37 plastomes from Bromeliaceae also found new lineage-specific rearrangements, including significant shift of IR boundary and large inversions in Tillandsioideae [58]. Many large repeats and rearrangements were also found in the four plastomes of *Juncus* (Juncaceae) [60]. Nevertheless, large-scale study about the general evolutionary pattern of plastome in the Poales as a whole is still lacking.

At present, more than 1000 plastomes of Poales were sequenced and deposited in the NCBI database (last access: October 10, 2022), but about 85% of them belong to a single family of Poaceae. Sixteen families are recognized in Poales, which could be divided into 5 groups: (1) early-divergent grade (Bromeliaceae, Typhaceae, Rapateaceae); (2) cyperid clade (Thurniaceae, (Cyperaceae, Juncaceae)); (3) xyrid grade (Mayacaceae, Eriocaulaceae, Xyridaceae); (4) restiid clade (Anarthriaceae, (Centrolepidaceae, Restionaceae)); and (5) graminid clade (Flagellariaceae, (Joinvilleaceae, (Ecdeiocoleaceae, Poaceae)) [11, 61, 62].

With more than 23,000 species, Poales diversified with extremely high diversity in terms of habits, morphologies, photosynthesis pathways, and pollination types, exhibiting a wide range of adaptations [49, 61, 63, 64]. Members of Poales exhibit all three photosynthetic pathways (C_3_, C_4_, and Crassulacean acid metabolism (CAM)), and species with C_4_ and CAM photosynthetic pathways are often adapted to exposed, hot, and dry environments [65]. CAM photosynthesis in Poales is restricted to Bromeliaceae, where it occurs in more than 40% of the species surveyed [66]. More than 6000 species of Poales have C_4_ photosynthesis, accounting for about 80% of the C_4_ plants in angiosperms, and are concentrated in Cyperaceae and Poaceae, each of which exhibit multiple origins of the C_4_ pathway [65, 67].

In addition, the ancestors of Poales appear to have been distributed in seasonally swampy/moist, highly infertile, and fire-prone habitats [49, 64]. This ancestral habitat was inherited by some extant families of Poales including Eriocaulaceae, Flagellariaceae, Juncaceae, Mayacaceae, Rapateaceae, Thurniaceae, Typhaceae, and Xyridaceae [49]. Recently, positive selection and photosynthesis-related adaptive mutations were detected in the evolution of the *rbcL* gene in several C_4_ plant groups including those species from the Poales [53, 68, 69]. Whether there were any genes involved in the adaptive evolution to the swampy habit and the photosynthesis-related adaptive evolution at the whole plastome level remain unexplored [13].

In our previous study, we sequenced 60 plastomes from 16 families of Poales [61]. Based on this dataset and publicly available data, we further developed a dataset of 93 plastomes of Poales (including 5 incomplete ones) representing 16 families and phylogenetic diversity of Poales at the family level. Our purpose is (1) to reveal the general pattern of plastomic variation in Poales; (2) to investigate the molecular mechanism underlying the variation; and (3) to explore the potential role of plastome variation may have played in the adaptive evolution of Poales. In short, we unfolded the unprecedented plastomic variation in the Poales and depicted their evolutionary trajectories. We found that the plastomic variations showed a pattern of “small-large-moderate” in the evolution and diversification of Poales, possibly contributing to the adaptation of its species to a wide range of habitats. Our study thus provides a comprehensive insight into the unusual variation of plastome in Poales in particular and for the understanding of the plastid evolution in angiosperms in general.

## Results

### Dynamic configuration and characteristics of plastomes in Poales

**Fig. 1:**
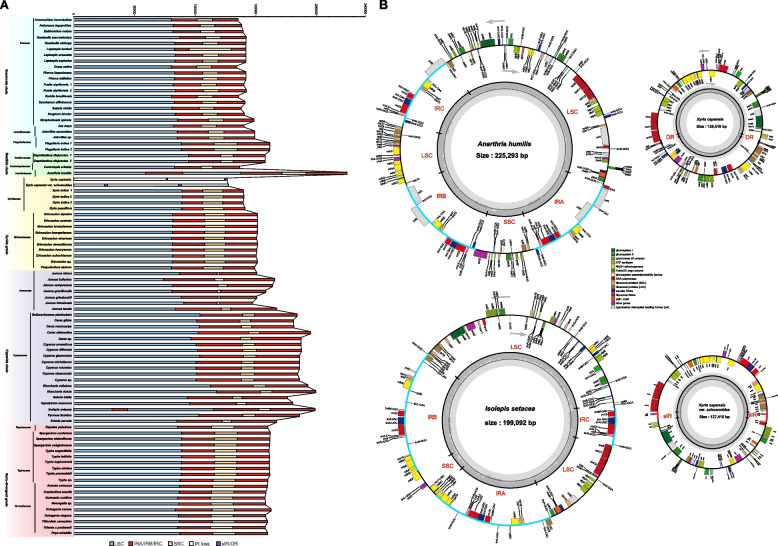
Characterization of the plastomes of Poales. **A** Size and structure of the plastomes. LSC: large single-copy region, IRa/IRb/IRc: inverted repeat region, SSC: small single-copy region, IR loss: lost one IR copy, sIR/DR: short inverted repeat/direct repeat. Different color block represents different region. **B** Four unconventional plastomic maps of Poales. Different gene blocks colored to indicate different functional groups. The dark gray bar in the center of each plot represents the GC content of plastom. Four regions of plastome were indicated in the circular map, as well as the ~ 3.3 kb short inverted repeat (sIR) and ~ 1.7 kb small direct repeat (DR)

Our dataset of 93 plastomes represents all the 16 families of Poales (Additional file 1: Table S1). Among the 60 sequenced plastomes in our previous study [61], the average sequencing depth of 55 complete plastomes ranged from 165.77 × (*Anarthria humilis*) to 7645.98 × (*Leptaspis urceolata*) (Additional file 1: Table S2), average at 1411.11 × . However, we were not able to acquire the complete plastome sequences for five species of Thurniaceae, Mayacaceae, and Ecdeiocoleaceae, but with only a few number of scaffolds assembled. The plastomes of Poales could also have multiple different configurations, and such plastomic alternations are mainly found in the families of Anarthriaceae, Cyperaceae, Juncaceae, Restionaceae, and Xyridaceae (Additional file 1: Table S1; Additional file 2: Fig. S1). After careful examination, all of the analyzed plastomes of Poales were assembled to have a typical quadripartite structure except for three species, *Anarthria humilis, Isolepis setacea*, and *Xyris capensis* (plus *Xyris capensis* var. *schoenoides*) (see details in the next section) (Fig. 1 and Additional file 2: Fig. S2).

Extensive variation in the genomic size and GC content of plastomes were found in the Poales (Fig. 1A and Additional file 1: Table S2). The largest plastome within the Poales, also the largest one within the monocots reported to date, was from the *Anarthria humilis* (Anarthriaceae) of 225,293 bp and approximately twice of the smallest one at 126,519 bp in *Xyris capensis* (Xyridaceae) (Fig. 1B). Similar difference was observed for the size of the LSC (117,896 bp in *Eleocharis dulcis* (Cyperaceae) vs. 74,401 bp in *Juncus bufonius* (Juncaceae)) and IR regions (41,905 bp in *Carex siderostica* (Cyperaceae) vs. 16,991 bp in *Xyris indica* (Xyridaceae)), while the SSC region showed a much greater variation from 34,770 bp in *Leptaspis banksii* (Poaceae) to just 1961 bp in *Juncus grisebachii* (Juncaceae) and about 17.7-fold difference (Additional file 1: Table S2). The general trend of size variation was that the early-divergent grade of Poales, i.e., Bromeliaceae, Typhaceae, and Rapateaceae, were relatively conserved in evolution, and the subsequently diverging groups (the cyperid, xyrid, and restiid) showed a high degree of variations, and the finally diverging graminid clade had a moderate variation (Fig. 1A).

The GC content of plastomes also varied in a large range from the lowest of 31.2% in *Mayaca fluviatilis* (Mayacaeae) to the highest of 39.1% in two *Guaduella* species (Poaceae). Like the observed pattern of genome size variation, the GC content was relatively conserved and with a level of 37.4%, 36.7%, and 36.8% on average for Bromeliaceae, Typhaceae, and Rapateaceae (Additional file 1: Table S2; Additional file 2: Fig. S3), respectively. Then the GC content was generally decreased along the diversification of the cyperid, xyrid, and restiid, especially for Mayacaceae at 31.2%, Cyperaceae at 33.6%, and Xyridaceae at 33.5% on average. Finally, the GC content rose in the graminid to reach the highest of 39.1% in Poaceae.

### Expansion/contraction of IR and different IR types

**Fig. 2:**
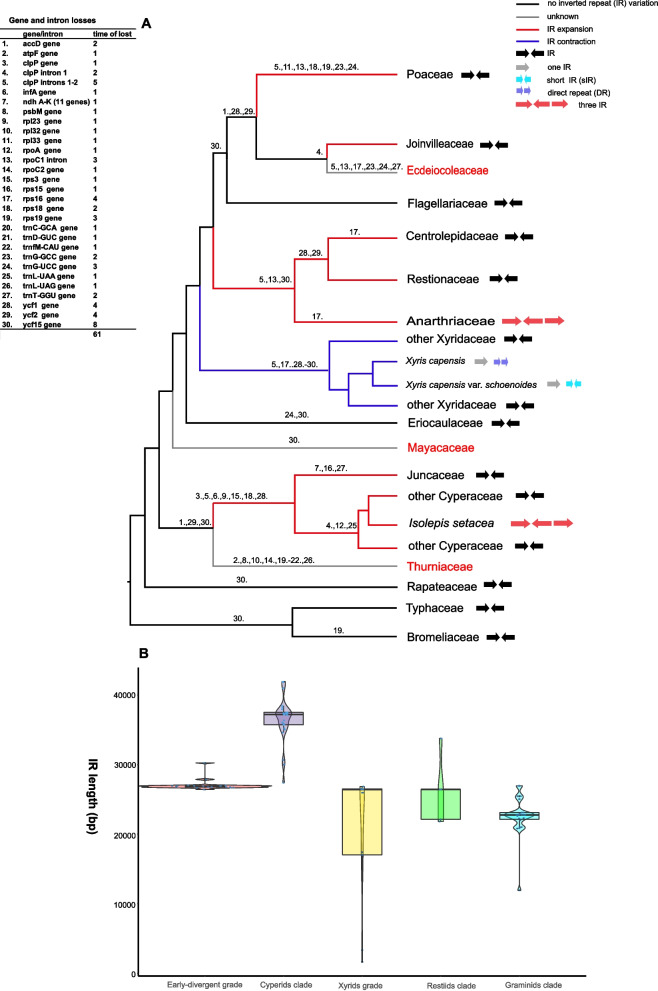
Gene/loss content and IR variation. **A** ML phylogram showing gene/intron losses, IR types, and IR variation in Poales plastomes. The tree was based on our phylogenetic analysis of plastomes of Poales [61]. **B** Variation of IR length in each clade of Poales, boxes show the first and third quartiles and the horizontal line represents the median values of each clade

**Fig. 3:**
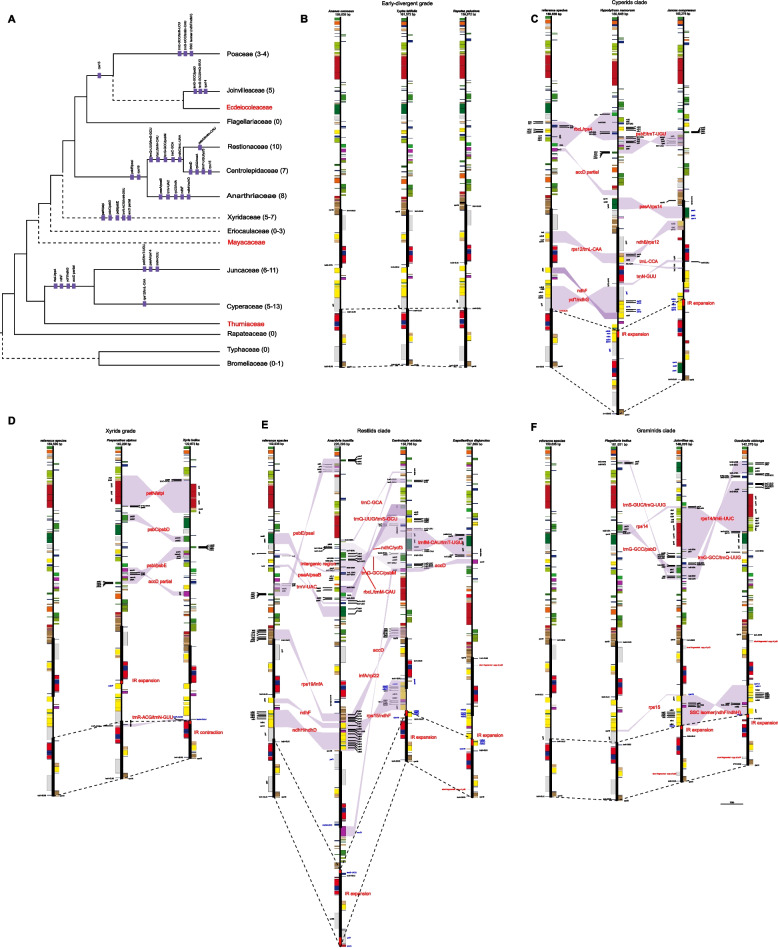
Structural evolution of plastomes in each clade of Poales. The plastomic maps show the inversion trend and variation in the IR boundary. Families mark in red text with incomplete plastomes. **A** The inversions are marked on the phylogenetic tree based on our previous work [61], numbers in parentheses mean the estimated range of inversions. **B** The plastomic structural evolution of the early-divergent grade. **C** The plastomic structural evolution of cyperid clade. **D** The plastomic structural evolution of the xyrid grade. **E** The plastomic structural evolution of restiid clade. **F** The plastomic structural evolution of graminid clade. Purple shades represent inversion endpoints, IR variation is indicated by connecting IR regions with dashed lines, black bars represent IR regions, red arrows that indicate the direction of IR shift, and the genes are influenced by the IR shift with blue text. The short fragmented copy of genes is listed in red

Although the IR region of plastome is generally conserved in evolution, both the massive expansion and contraction of IR were recovered in the Poales, and in the extreme cases even the complete loss of one IR copy or the gain of a third copy of IR occurred (Figs. 2A and 3; Additional file 2: Fig. S2). The plastomes of Anarthriaceae and Cyperaceae experienced the greatest degree of IR expansion in the Poales. The IR of Anarthriaceae was expanded to include both the LSC and SSC regions with the whole *ycf1* and *trnG-UCC* genes located in it, as well as partial *accD* and *rpoA* sequences. In the Cyperaceae, expansion of IR also had the whole *ycf1* from the SSC and even with the *rps15* and *ndhA*, *G*, *H*, and *I* genes being included (Fig. 2A; Additional file 2: Fig. S2). Eventually, the SSC region just had only seven genes retained. More strikingly, three IR copies were both found in two species of *Anarthria humilis* (Anarthriaceae) and *Isolepis setacea* (Cyperaceae) (Fig. 1B). The IRa and IRb were equal in length and encoded the same genes both in the two species, while the third copy defined as IRc was shorter with fewer number of genes. In *Anarthria humilis*, the IRa/IRb was expanded to 33,752 bp and IRc was slightly shorter of 26,551 bp due to the loss of *ycf1* gene. The orientation of IRc was the same as IRa. And IR expansion and the gain of a third IR copy made *Anarthria humilis* being the largest known plastome in the monocots. In *Isolepis setacea*, the IRa/IRb was further expanded to 37,112 bp; however, the 11,339 bp IRc only contained a core set of four rRNA genes (*4.5S*, *5S*, *16S*, and *23S*) and four tRNA genes (*trnR-ACG*, *trnA-UGC*, *trnI-GAU*, and *trnfM-CAU*) (Figs. 1B and 2A; Additional file 1: Table S2).

On the other hand, IR in the Xyridaceae species was contracted with the original genes of *trnR-ACG*, *trnN-GUU*, and *ycf1* all being relocated in the SSC region. Moreover, only one complete IR copy occurred in *Xyris capensis* and *X. capensis* var. *schoenoides*. A pair of sIR of 3343 bp in *X. capensis* var. *schoenoides* and DR of 1650 bp in *X. capensis* was found, and both of which encoded two genes of *infA* and *rpl36*, likely playing a role like typical IRa/IRb (Fig. 1B; Additional file 2: Fig. S4). In addition, the plastome assemblies of *Anarthria humilis, Isolepis setacea, Xyris capensis*, and *X.* *capensis var. schoenoides* were selected for PCR verification, and the sequencing results supported the presence of three IR copies, sIR and DR in these genomes, respectively (Additional file 1: Table S3 and Additional file 2: Fig. S4).

For the remaining families, we found that the plastomes of Bromeliaceae, Rapateaceae, and Typhaceae were relatively conserved in the IR boundary, as well as Flagellariaceae of the graminid clade. However, the IR of Joinvilleaceae and Poaceae was expanded to the SSC region with the *rps15* gene being included. In short, the IR expansion/contraction and gain/loss (Fig. 2B) echoed the trend of variation pattern in plastome size during the evolution of Poales as described above.

### Multiple gene and intron loss

Comparison of the 93 plastomes of Poales showed that they encoded a unique set of 96 to114 genes, including 63 to 80 protein-coding, 25 to 30 tRNA, and 4 rRNA ones (Additional file 1: Table S2). However, with the exception of 34 genes (*atp* (*A*, *B*, *E*, *H*, and *I*), *ccsA*, *cemA*, *matK*, *psaI*, *psb* (*A*, *C*, *D*, *E*, *F*, *I*, *J*, *K*, *L*, and *Z*), *rbcL*, *rpl16*, *rpoB*, *rps* (*2* and *4*), *trn* (*E-UUC*, *F-GAA*, *Q-UUG*, *S-GCU*, *S-GGA*, *S-UGA*, *T-UGU*, and *V-UAC*), *ycf* (*3* and *4*)), the remaining ones all experienced some kinds of sequence duplication, degradation to being short fragmented copy or gene and intron loss in certain plastomes (Fig. 2A; Additional file 2: Fig S5).

At least one loss event was observed for the 38 genes and introns in the Poales, and the most frequent lost was *ycf15* with 8 times, followed by 2 introns of *clpP* with 5 times and *rps16*, *ycf1*, and *ycf2* with 4 times. The gene/intron loss events were mostly found in the plastomes of cyperid, xyrid, and restiid. Moreover, loss or degradation of all the *ndh* genes with just short fragments remained were only observed in certain species of Juncaceae (Fig. 2A). By contrast, only one gene loss was found in the plastome of Flagellariaceae as the early-divergent family of the graminid clade. However, multiple gene and intron loss occurred in the remaining three families, particularly Poaceae. Gene duplication was also found in addition to the 17 common genes in the IR region, a total of 51 genes with two or more copies were found (Additional file 2: Fig. S5).

### Widespread occurrence of inversions

We further built a dataset of 88 complete plastomes to separately perform synteny analysis with representative species from each family with the least variation for illustration of inversions, a major structural rearrangement in the plastome evolution. Inversions were found in more than one third of the families in the Poales, and the majority of them were larger than 1 kb and found in the LSC region (Fig. 3; Additional file 1: Table S4). The early-divergent families were conserved with no inversion detected as compared to the typical plastome structure of flowering plants and with *Ananas comosus* used as the reference here, except for one hybrid species of Bromeliaceae (*Vriesea* x *poelmanii*), which had a ~ 28 kb inversion from *psbD* to *accD* (Fig. 3A, B; Additional file 1: Table S4).

Within the cyperid clade, the species of Cyperaceae and Juncaceae had 5–13 and 6–11 inversions, respectively. In Cyperaceae, most species shared six inversions, ranging from ~ 1 to ~ 6–10 kb. In Juncaceae, the occurrence of inversions was more diversified and most species only shared two inversions of ~ 1 kb and ~ 5–6 kb in size (Additional file 1: Table S4). At the family level, Cyperaceae and Juncaceae shared four inversions, and specifically had one inversion and three inversions (Fig. 3A, C), respectively.

In the xyrid grade, the plastomes of Eriocaulaceae had three inversions at most (Additional file 1: Table S4). However, the plastome of *Paepanathus alpinus* (Eriocaulaceae) showed great collinearity with that of *Ananas comosus* and no inversion was detected. Five to seven inversions were identified in Xyridaceae, and most species of this family had common five ones, with the largest one being ~ 17–20 kb. At the family level, Eriocaulaceae and Xyridaceae did not have shared inversions with only five unique inversions found in Xyridaceae (Fig. 3A, D).

There were eight, seven, and ten inversions identified in the three families of Anarthriaceae, Centrolepidaceae and Restionaceae of the restiid clade, respectively (Additional file 1: Table S4). Two common inversions were shared by all the three families. Moreover, the families of Centrolepidaceae and Restionaceae shared additional five inversions and had four and one unique inversions, respectively (Fig. 3A, E).

In the graminid clade, Joinvilleaceae and Poaceae contained five and three to four inversions, respectively. All the analyzed Poaceae species contained the three well-documented inversions (~ 28 kb, ~ 6 kb, and < 1 kb) in the previous study [55]. The *rpl2*/partial *accD* (~ 30 kb) inversion was only found in the early-divergent grass of *Guaduella macrostachys*. The occurrence of inversions in Poaceae was different from the pattern observed in the remaining families of Poales and the diversity was limited with the majority of inversions shared by all sampled grasses, indicating that they arose prior to the origin of this family (Additional file 1: Table S4). In addition, Flagellariaceae was relatively conserved without inversion detected. Although the representative species of Joinvilleaceae and Poaceae shared the *rps*15 inversion, they also exhibited three and two unique inversions, respectively (Fig. 3A, F).

## Abundant repeats and heterogeneity of substitution rate

**Fig. 4:**
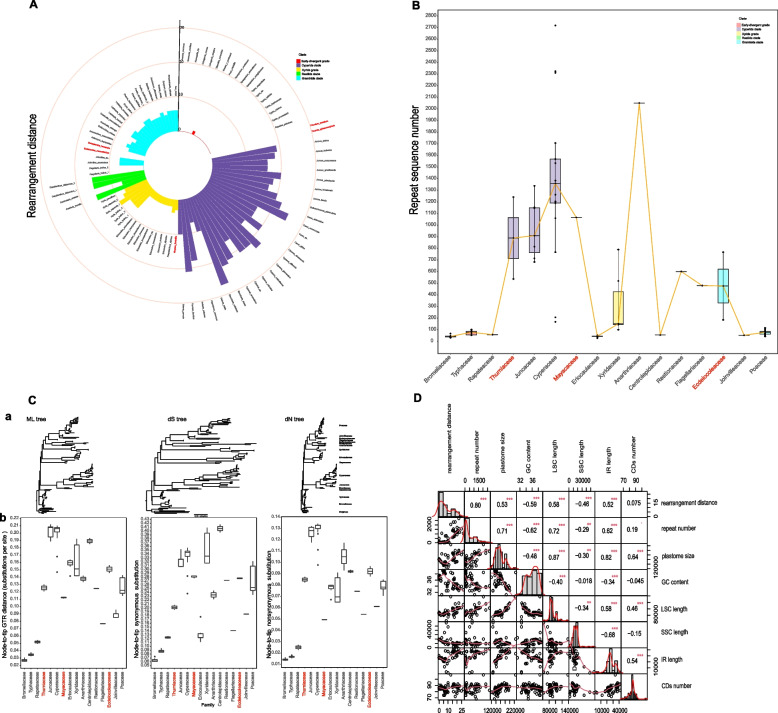
Variation of repeat sequence number, rearrangement distance and substitution rate, and multivariable pairwise correlation analysis of plastomes in Poales. **A** The rearrangement distance of 93 plastomes in Poales. Different color represents different clade of Poales. **B** The variation of repeat sequence number of each family in Poales. The orange solid line represents the median values of each family. **C** Substitution rates in the 80PG-half matrix based on maximum likelihood method of Poales plastomes. (a) Branch lengths are in the field of substitution rates in ML tree, synonymous (dS) and nonsynonymous (dN) tree. All trees are in the same scale. Bar, 0.05 substitutions per site. (b) Comparison of the intra-ordinal plastid branch lengths among the Poales families, as estimated by root-to-tip patristic distances, from the common ancestor of Poales to each sampled tip (unpartitioned GTR-distance from RaxML analysis of the 80PG-half matrix). **D** Multivariate correlation and frequency distribution. The upper right corner represents the correlation coefficient and its significance between the two variables, the lower left corner represents the scatter plot of the correlation between the two variables, and bar graph on the diagonal represents the distribution of the quantity of the corresponding variable, the curve represents the frequency distribution plot of the corresponding variable. Asterisk means significant

With large amounts of inversions observed, we calculated the genomic rearrangement distance. As expected, the largest distance was found in the cyperid clade, followed by the restiid clade, while the smallest one occurred in the early-divergent grade of Poales (Fig. 4A). We further detected repeats (≥ 30 bp) in the 93 plastomes. Among families, Cyperaceae had the largest number of repeats, with the maximum of 2718 in *Carex siderostica* and Bromeliaceae and Eriocaulaceae had the least number of repeats and both under 50. At the clade level, the cyperid clade had the largest number of repeats as well as the largest variation in the number from 168 to 2718, while the early-divergent grade had the least (Fig. 4B). In addition, the majority of identified repeats ranged in size from 30 bp to 1 kb, and a few repeats larger than 1 kb were only found in families of Anarthriaceae, Cyperaceae, Juncaceae, Mayacaceae, Poaceae, Restionaceae, and Xyridaceae (Additional file 2: Fig. S6).

Our plastome-based phylogenetic tree of Poales [11, 49, 61] possessed a combination of short and long branch lengths, indicating heterogeneous molecular evolutionary rates among families (Additional file 2: Fig S7). Being slow in the early-divergent grade, the substitution rate in the Poales gradually increased from the early-divergent grade to the restiid clade and reached the highest in the Juncaceae of the cyperid clade, and afterwards decreased in the graminid clade. Dividing the substitution rate into synonymous and non-synonymous, we also obtained a similar trend of variation (Fig. 4C). We further used three clock models (global clock, local clock, and clockless) to investigate the shifts in the rate of nucleotide substitution across Poales. The clockless model was the best and the local clock model was better than the global clock model according to the corrected Akaike information criterion (AICc) (Additional file 1: Table S5). Under the local clock model, the early-divergent grade had the lowest substitution rate as inferred from the branch lengths of phylogenetic trees above, while the highest rate in the intermediate lineages (cyperid clade, xyrid grade, and restiid clade) (Additional file 1: Table S5), exhibiting a 3.5-fold difference. These results clearly demonstrated the sharp shifts in the rate of nucleotide substitution during the evolution of Poales.

### Correlations between plastomic characters and genomic variation

To investigate the molecular mechanism underlying the plastomic variation in Poales, we selected eight pairs of variables of 93 plastomes for multivariate correlation analysis and found that most of them were significantly correlated with each other (Fig. 4D). We considered that the correlation coefficient (*r*) at |*r*|≥ 0.8, 0.5 ≤|*r*|< 0.8, and |*r*|< 0.5 represented strong, moderate, and weak correlation, respectively. The rearrangement distance was found to be positively correlated with the repeat number (*r* = 0.80), as well as with other variables such as genome size, LSC size, and IR size with *r* at 0.53, 0.58, and 0.52, respectively. The repeat number was also moderately correlated with the genome size, LSC size, and IR size with *r* at 0.71, 0.72, and 0.62, respectively (Fig. 4D; Additional file 2: Fig. S8). In addition, the inversion numbers showed positive correlation with the repeat numbers (*r* = 0.79), as well as with the rearrangement distance (*r* = 0.95) as expected (Additional file 2: Fig. S9). On the other hand, negative correlations were found in the GC content between rearrangement distance (*r* =  − 0.59) and repeat number (*r* =  − 0.62). And the SSC size was negatively correlated with the IR size (*r* =  − 0.68).

### Positive selection of genes in plastome

**Fig. 5:**
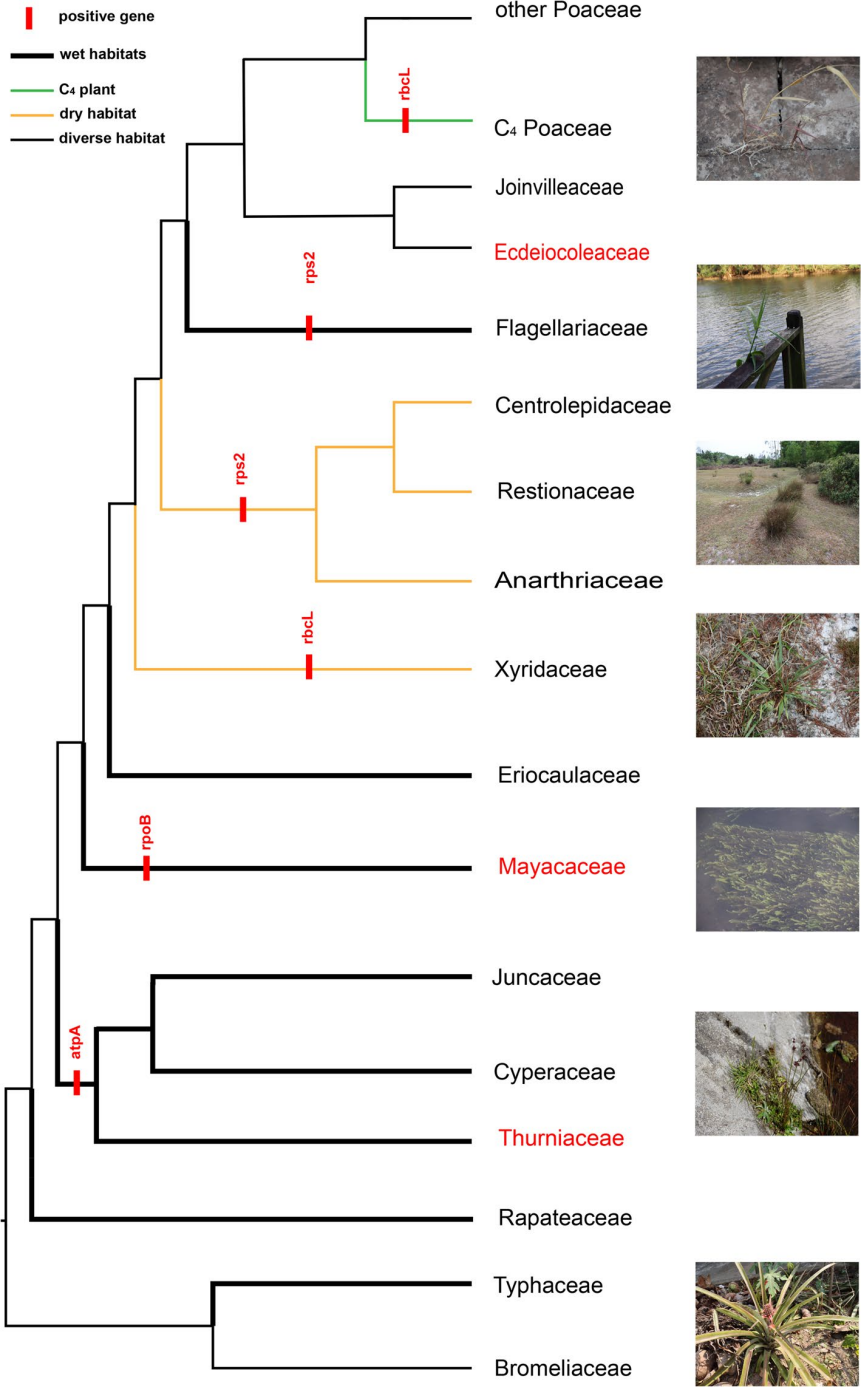
Selection pressure analyses based on the branch-site model. Positively selected genes of branches are marked in red text with red rectangle. Different line segments of phylogenetic tree are used to mark the habitats of each family and photosynthetic pathways of Poaceae in Poales. On the right are pictures of the habitat of Poales. The phylogenetic tree based on our previous work [50]. Habitat of *Setaria viridis*, *Flagellaria indica*, *Dapsilanthus disjunctus*, *Xyris indica*, *Mayaca fluviatilis*, *Juncus* sp., and *Ananas comosus* (from up to bottom)

To detect the potential selection of protein-coding genes in the plastomes, we firstly used site-specific model and branch-site model to analyze 34 genes shared by all sampled 93 Poales species. Based on the site-specific model, we identified 8 genes (*atpA*, *psbK*, *rbcL*, *rpl22*, *rpoB*, *rps2*, *rps7*, and *ycf3*) showing positive selection signals (Table 1). To further reveal in which families the positive selection of these 8 genes occurred, we performed selection analyses with the branch-site model on them. We found that *atpA* and *rpoB* experienced positive selection in the cyperid clade (*P* = 0.013) and Mayacaceae (*P* = 0.017), respectively. Intriguingly, the *rbcL* gene was under positive selection in the Xyridaceae and the C_4_ clade of Poaceae, with five positively selected (*P* = 4.4E − 07) and one positively selected (*P* = 0.012), respectively. The *rps2* gene was positively selected parallelly in the Flagellariaceae (*P* = 0.016) and restiid clade site (*P* = 0.014), respectively (Fig. 5; Additional file 2: Table S6). In addition, we also estimated the dN/dS ratio on each branch of the phylogenetic trees of 29 genes with loss events in certain plastomes. We found that all of them with the exception of *ycf2* displayed an average dN/dS ratio < 1, indicating they were undergoing purifying selection. Moreover, the closely related lineages of those experiencing gene loss within the same family tended to have a larger dN/dS ratio than those of sister families without gene loss (Additional file 1: Table S7).

**Table 1:** Likelihood ratio test (LTR) of the variable *ω* ratio under different models

Gene	Comparisons	InL1	InL2	np1	np2	2ΔL	*df*	*P*-value
*atpA*	M0vsM3	− 16,937	− 16,174	211	215	1525.05	4	0
	M1vsM2	− 16,320	− 16,301	212	214	37.6083	2	6.81E − 09
	M7vsM8	− 16,191	− 16,154	212	214	73.2202	2	1.11E − 16
*psbK*	M0vsM3	− 2042.2	− 1953.3	211	215	177.742	4	0
	M1vsM2	− 1969.1	− 1962.1	212	214	13.9514	2	0.0009343
	M7vsM8	− 1963.8	− 1952.3	212	214	23.1282	2	9.50E − 06
*rbcL*	M0vsM3	− 8470.6	− 8005.3	211	215	930.656	4	0
	M1vsM2	− 8092.5	− 8078.6	212	214	27.7414	2	9.46E − 07
	M7vsM8	− 8017.7	− 7997.7	212	214	40.1185	2	1.94E − 09
*rpl22*	M0vsM3	− 516.87	− 487.16	211	215	59.4117	4	3.86E − 12
	M1vsM2	− 496.97	− 491.09	212	214	11.7556	2	0.0028009
	M7vsM8	− 496.55	− 487.74	212	214	17.6208	2	0.0001492
*rpoB*	M0vsM3	− 6799.1	− 6579.9	211	215	438.402	4	0
	M1vsM2	− 6641.3	− 6631.8	212	214	18.9368	2	7.73E − 05
	M7vsM8	− 6585.8	− 6573	212	214	25.6444	2	2.70E − 06
*rps2*	M0vsM3	− 3175.9	− 3110.4	211	215	130.973	4	0
	M1vsM2	− 3130.3	− 3124.7	212	214	11.2962	2	0.0035241
	M7vsM8	− 3118.9	− 3108.1	212	214	21.5384	2	2.10E − 05
*rps7*	M0vsM3	− 837.15	− 803.96	211	215	66.3752	4	1.32E − 13
	M1vsM2	− 808.53	− 804.83	212	214	7.40333	2	0.0246824
	M7vsM8	− 812.13	− 806.16	212	214	11.9333	2	0.0025629
*ycf3*	M0vsM3	− 1398.6	− 1312.8	211	215	171.615	4	0
	M1vsM2	− 1316.9	− 1313.9	212	214	6.02576	2	0.04915
	M7vsM8	− 1321.7	− 1315.1	212	214	13.189	2	0.0013679

A *P*-value equal to 0 means that the value is infinitesimally close to 0

## Discussion

### Diverse patterns of plastome variation in Poales

With the rapid development of sequencing technology, growing numbers of plant plastomes have been sequenced. However, a few studies have been conducted for an order at the family level [70, 71], with recent studies expanding coverage across all families of monocots [11] and angiosperms [72] while mainly focusing on phylogenetic relationships rather than plastomic evolution. Most studies have focused on the plastomic evolution at the familial and generic level, such as in algae and non-photosynthetic flowering plants [22, 36, 73]. The estimated 23,000 species of Poales are all photosynthetic autotrophic plants including four carnivorous species (two species of *Brocchinia*, *Catopsis berteroniana*, and *Paepalanthus bromelioides*) with diversified photosynthetic pathways distributed in various habitats [64]. Previous studies indicated certain variation in the plastomes of Poales but generally focused on individual families [39, 57–59]. Here, we expanded sampling of Poales representing all the 16 families of Poales, revealing diverse varying patterns among families from genome size, gene content to the GC content. The *Anarthria humilis* (Anarthriaceae, restiid clade) has the largest plastome within the monocots reported to date, being of ~ 225 kb, just 18 kb smaller than the largest one in angiosperms (*Pelargonium transvaalense*, 243 kb) [19]. This genome is about twice as large as the smallest one within Poales, i.e., *Xyris capensis* of ~ 127 kb (Xyridaceae, xyrid grade). At the family level, Cyperaceae has larger plastomes, averaging at ~ 186 kb and about 26 kb larger than the typical ones [39]. The plastomes of the other families were all in the typical range of size but with its own specificity for each family. The observed variation of plastome size mainly came from the groups of cyperid, restiid, and xyrid while moderate variation for the graminid clade and least for the early-divergent grade of Poales.

The plastomic GC content also varied among different families of Poales. The GC content was nearly the same at 37.0% for different species from the early-divergent grade of Poales and decreased in the cyperid, restiid, and xyrid, reaching the lowest value of 31.6% in Mayacaceae. Finally, the GC content was increased in the graminid clade with the highest of 39.1% found in Poaceae. The GC content of sequenced angiosperm plastomes ranged from 22.67 to 43.20% [74]. The highest and lowest GC content of Poales differed by 7.5%, showing a relatively large variation within an order of angiosperms. Moreover, this trend of variation was parallel to that observed in the GC content of nuclear genome [75] also with the highest level found in Poaceae (Additional file 1: Table S2).

The loss of gene/intron events of Poales were also diverse and species-specific. A total of 40 gene/intron that were documented to be lost with the most frequent lost genes were *rps16*, *ycf1*, *ycf2*, and *ycf15*. In addition, previously reported unusual loss of *accD* gene and introns of *clpP* and *rpoC1* actually occurred multiple times in Poales [42–44, 55–57], further strengthening the distinct pattern of plastomic variation in Poales. These events almost included all types of gene/intron loss previously reported in the photosynthetic plants [4, 24, 76]. It was noted that gene duplications were also diverse in Poales and many such as *infA* and *rps16* were not found before [39]. Gene/intron loss and gene duplication occurred most frequently in the groups of cyperid, restiid, and xyrid of Poales, followed by the graminid clade, and almost never found in the early-divergent grade.

There was an unexpected variation of IR types, as well as extreme IR expansion/contraction in Poales. The expansion of IR occurred in nine families, mainly extending to the SSC region (Figs. 2 and 3). In comparison, the contraction of IR was only found in Xyridaceae, even with the complete loss of one IR copy in two plastomes of *Xyris capensis* and *X*. *capensis* var. *schoenoides* and just having a small DR and sIR, respectively. IR loss has been reported in many different lineages of angiosperms [20, 23, 24, 26, 27, 32, 77], and our observation added Poales to this list. In addition, IR loss could lead to the accumulation of new small repeats and the existence of small DR and sIR may replace the function of the original IR [26, 34, 35, 37, 77]. Two species *(Anarthria humilis* and *Isolepis setacea*) possessed three IR copies, which was one of the reasons for the largest plastome within the monocots. Previously, three IR copies were only found in the green algal genus *Chamaetrichon* [36], and to the best of our knowledge, this was the first such report in angiosperms, further underlining the diversity and complexity of plastomes in Poales. The number of detected inversions in the individual plastome of Poales ranged from 0 to 13, with the most number observed in *Carex neurocarpa*. The length of inversions ranged from ~ 110 bp to ~ 33 kb with the longest one found in *Juncus tenuis*. However, there was a few inversions shared by different species and families in Poales, and the majority of shared inversions were found in the Poaceae. Previous study suggested unprecedented structural heteroplasmy in two *Eleocharis* species [39], we have also observed similar phenomena in certain species here, particularly those that presented challenges in obtaining a circular plastome.

### The small-large-moderate trend of variation in the plastome evolution of Poales

The plastomes of Poales showed extensive variation from genome size and gene content to the GC content. Moreover, a trend of small-large-moderate variation pattern during the diversification of Poales was revealed, with relatively conserved in the early-divergent grade, large variation in the intermediate groups (cyperid, restiid, and xyrid) and moderate variation in the graminid clade. This trend was also observed for the genomic rearrangements, including IR variation and inversions. With respect to the molecular evolution rate, a heterogeneous pattern was observed [11, 48, 50–52], exhibiting a similar trend of small-large-moderate variation.

In addition, we found the correlation between the different characteristics of plastomes in Poales (Fig. 4D). There were many moderate (100–999 bp) and large (> 1 kb) repeat sequences in the plastomes of Poales, which on the one hand made the genome larger and on the other hand led to genomic rearrangements (Additional file 2: Fig. S6). Homologous recombination between repeats could cause inversions and other rearrangements [34]. As expected, we found a strong correlation between repeat number and inversions, inversions and rearrangements in the plastomes of Poales (Additional file 2: Fig. S7).

### Adaptive evolution of plastomes

Gene loss is frequent in Poales, particularly the loss of *ndh* genes in some species of Juncaceae [60]. The loss of *ndh* genes reported previously mostly occurred in the non-autotrophic plants [2] such as *Epijagus virginiana* [78], *Cuscuta* [15, 79], *Hypopitys monotropa* [4], and *Triantha occidentalis* [71, 80]. The loss of *ndh* genes were also reported in the photoautotrophic plants, such as *Selaginella* [21, 44], *Carnegiea gigantea* [32], and some carnivorous species of Lentibulariaceae [81], particularly in the submersed plants such as those in Hydrocharitaceae and seagrasses [71, 82]. These genes are mainly involved in photosynthesis [82] and may have been transferred to the nucleus in the photoautotrophic plants [83]. It has been suggested that their loss may be related to environmental adaptation for a special seagrass environment [66], or working together with direct repeat for arid habit [23]. In fact, the majority of plants reported with the loss of *ndh* are hydrophytes [71, 82], and the Juncaceae plants are also hydrophytes [64]. We thus speculate that the dynamic change of *ndh* gene here could be an adaptation to the swampy environment although Cyperaceae may adopt a different evolutionary pathway. During the transition from an autotrophic to a non-photosynthetic parasitic lifestyle, the plastome will also undergo gradual relaxation of negative selection [84]. Furthermore, our study suggests that closely related lineages of those experiencing gene loss are evolving under more relaxed purifying selection, a phenomenon similar to the trajectory of plastomic evolution during the transition from autotrophy to a non-photosynthetic parasitic lifestyle. As a result, we speculated that the relaxed purifying selection may be an adaptation to the environment [84]. Variation in GC content was thought to affect multiple processes such as mutational bias and recombination and which are in turn related to genome function and ecological fitness of organisms [74, 75]. The high GC content of nuclear genome in grasses was proposed as an adaptation to stressful environments of seasonally cold and dry climates [75]. A similar phenomenon was also observed in the plastome with higher GC content for the Poaceae and its sister families. The cyperid clade and the xyrid grade have the lowest GC content in Poales and species in both of them are mainly distributed in wetland or swampy habitats. In addition, accelerated substitution rate, as demonstrated in certain families of Poales, could also be an indication of adaptive evolution [48].

Furthermore, we found positive selection signals for eight plastid genes including *rpl22*, *rps2*, *rps7*, *rpoB*, *ycf3*, *psbK*, *atpA*, and *rbcL*. Four of them (*atpA*, *rbcL*, *rpoB*, and *rps2*) were also under selection by the branch-site model. ATP synthase is usually the product of two genetic systems and is required for photosynthesis in plants [85] and *atpA* was also positively selected in seagrass (*Zostera*) [66]. The *rps2* encodes ribosomal proteins S2 [86] and adaptive evolution of it involved protein synthesis in angiosperm plastids [87]. We found positive selection of three genes (*atpA*, *rpoB*, and *rps2*) in different clades or families, maybe related to their adaptation to the different environment. The *rbcL* gene plays a significant role as a regulator of photosynthetic electron transport, and it encodes the large subunit of RuBisCO [88]. Moreover, this gene was frequently under positive selection in land plants and underwent adaptive evolution [53, 68, 69, 89, 90]. In Xyridaceae, it may represent an adaptation to the seasonal dry habitat, while the positive selection in the C_4_ clade of Poaceae may be related to the C_3_–C_4_ photosynthetic transition.

In all, four out of the eight positively selected genes are all related to photosynthesis. Poales includes all three known pathways of photosynthesis with the origin of C_4_ occurred between 30 and 18 Mya in Poaceae [67, 91, 92] and between 19.6 and 10.1 Mya in Cyperaceae [93], and CAM between 16.2 and 8.1 Mya in Bromeliaceae [94], coinciding with increasing aridification, warm, and falling atmospheric CO_2_ concentrations [94, 95]. The transformations of photosynthesis pathways undoubtfully contributed to their adaptability to the changing environment [64, 92]. Therefore, Poales becomes the third largest order of angiosperms [63] and the variation and evolution of its plastomes may have played an important role. Further studies of more plastomes from species of different photosynthetic pathways would enhance our understanding of the relationship between the plastomic evolution and adaptability in angiosperms.

## Conclusions

In this study, we analyzed a dataset of 93 plastomes of Poales and revealed that the pattern of plastomic variation at the family level showed diverse patterns, mainly reflected in the genome size, GC content, different IR types, IR shift, heteroplasmy of genome structure and inversions. Interestingly, the plastomic variation exhibited a trend of “small-large-moderate” during the evolution of Poales, which is relatively conserved in the early-divergent grade, large variation in the intermediate groups (cyperid clade, restiid clade, and xyrid grade), and moderate variation in the graminid clade. The structural variation is closely related to the number of repetitive sequences found in the plastomes of Poales. Moreover, the plastomic substitution rate is also heterogeneous among Poales, showing a similar trend of “small-moderate-large” variation. Extensive variation of plastomes in Poales may be related to the adaptive evolution to the changing climate and the multiple photosynthetic pathway transitions. This study provides new insights into the complex architecture evolution pattern in one of the most diversified orders of plants and the adaptive evolution of plastomes in angiosperms.

## Methods

### Plastome assembly evaluation and PCR validation

The plastomes of Poales analyzed in this study included 60 from our previous study (Additional file 2: Table S1) [61] and 33 downloaded from GenBank (https://www.ncbi.nlm.nih.gov/). Using fastp [96] to process raw data to get high-quality clean data in the default set. Among the 93 plastomes, 88 were complete and 5 were assembled with gaps, representing all the 16 families of Poales. The methods of sequencing, assembly, and annotation used for the 60 plastomes generated by us could be referred to our previous study [87]. The potential plastome configuration were inferred by Getorganelle [97]. We further used Bandage for visualization of these plastomes [98] and the python script evaluate_assembly_using_mapping.py (https://github.com/Kinggerm/GetOrganelle/tree/master/Utilities) to evaluate the assembly results. Our definition of gene loss was based on the combination of gene length and sequence similarity. When no similarity and length greater than 60% were detected in the whole genome, the gene is considered to be lost. Gene fragments with a certain degree of similarity (greater than 60%) to the normal genes, but with the stop codons appeared early in the open reading frames are defined as short fragmented/partial copy of genes. In addition, we selected four plastomes of *Anarthria humilis, Isolepis setacea, Xyris capensis*, and *X.* *capensis* var. *schoenoides* showing great structural variation to perform validation by polymerase chain reaction (PCR) and Sanger sequencing. The designed primers were provided in Additional file 1: Table S3. The plastome maps were drawn using OGDRAW v1.3.1 [99].

### Plastome features and repeat analysis

We calculated the characteristics of plastome in Geneious v9.1.4 [100], including the genome size, gene content, and GC content. The presence and loss of genes in the plastomes were drawn using the heat map function of TBTOOLS [101] and the gene group map. Dispersed repeats defined into three types of forward, reverse, and palindromic were identified using REPuter [102]. The hamming distance was set as 3 the maximum and minimal repeat size was 5000 bp and 30 bp, respectively.

### Collinearity and rearrangement distance

The plastome of *Ananas comosus* was chosen as the reference as its good collinearity to that of *Nicotiana tabacum*. With one IR region removed to avoid mistakes, 88 complete plastomes were separately analyzed for collinearity with that of *Ananas comosus* using progressiveMauve software [103]. The orientation of the locally collinear blocks (LCBs) was confirmed and marked by a (+ / −) sign, and a negative sign indicated the presence of an inversion. Compared with the reference, the number of LCBs in each plastomes was counted. Finally, the corresponding genomic rearrangement distances were calculated using GRIMM [104]. Based on the rearrangement distance, the species with the least plastomic structural variation in each family was selected for illustration. The inversion and the shift of the IR boundary was marked by manual on the map.

### Multivariable pairwise correlation analysis

To investigate the potential underlying mechanism of plastome variation in Poales, we selected eight variables of GC content, repeats number, genome length, LSC length, SSC length, IR length, rearrangement distance, and CDs number for analyses. The correlation analysis between any two variables were carried out by the R, and the results were visualized by the ggplot2 packages.

### Plastomic substitution rate and inference of rate changes

The sequences of concatenated 80 plastid protein-coding genes were trimmed dataset with using the Gblocks [105] with allowed gap with half (80PG-half matrix), and then inferred phylogenetic tree by RaxML [106] with 1000 replicates using the GTRGAMMA model. We included 99 individuals of Poales and 7 outgroup taxa (7 species of Commelinales and Zingiberales) (Additional file 2: table S8) for analyses. Sequence divergence for each branch in the tree was calculated by HYPHY v.2.2.4 [107]. With synonymous (dS) and nonsynonymous substitution rates (dN) in the MG94xHKY85 codon model. In order to more accurately test the molecular evolution rate changes across Poales, we chose baseml of PAML [108] for analysis. We selected three clock models for comparative analysis like previous studies [48, 109]. The global clock model was that assuming all Poales lineages had the same molecular evolution rate. The local clock model was that assuming specified branches had different evolutionary rates and other branches had the same one. In the clockless model, the rates of each branch were shifty. Our analysis used the GTR + Γ model and the 80PG-half matrix with the corresponding phylogenetic tree derived from it.

### Selective pressure analysis

We calculated the ratio (*ω*) of the non-synonymous substitution rate (dN) and the synonymous substitution rate (dS) to estimate the selection pressure with site-specific and branch-site model. The 34 protein-coding genes common to all 106 samples were selected and computed by the codeml program in PAML [108] in the site-specific and branch-site model, respectively, based on phylogenetic tree of 80PG-half matrix. The one single-gene matrices (Additional file 4) were aligned and then treated by Gblocks with allowed gap with half. The site-specific model parameters were set to model = 0, NSsites = 0, 1, 2, 3, 7, and 8, and seqtype = 0. The *P*-values of likelihood ratio tests (LRTs) were calculated for the following three pairs of models to identify positively selected genes (*p* < 0.05) including M0 (one-ratio) vs. M3 (discrete), M1 (near neutral) vs. M2 (positive selection), M7 (*β*) vs. M8 (*β* and *ω*). The branch-site was used to evaluate potential positive selection in the Xyridaceae, Flagellariaceae, Eriocaulaceae, Mayacaceae, Typhaceae, Rapateaceae, the cyperid clade, the restiid clade, and C_4_ clade of Poaceae that were respectively as the foreground branches. A neutral branch-site model (Model = 2, NSsites = 2, Fix_omega = 1, omega = 1) and an alternative model (Model = 2, NSsites = 2, Fix_omega = 0, omega = 2) were used, respectively. The *P*-values were calculated by right-tailed Chi-square test off on the difference of log-likelihood values between the two models with one degree of freedom. Moreover, BEB method [110] was performed to compute the posterior probabilities for amino acid sites potentially under positive selection. *P*-value < 0.05 and *ω* > 1 was of the gene defined under positively selected gene. The posterior probability > 0.95 for a site was defined as positively selected. We performed a selection pressure analysis for 29 lost genes with 5 genes yielding meaningless results so only the results of the analysis of 24 genes were obtained. The species did not have the gene were manually pruned from the phylogenetic tree inferred from all 106 species used as a reference tree. Employing the corresponding tree, one ratio (model = 0, NSsites = 0) and the free ratio model (model = 1, NSsites = 0) was employed to calculate dN, dS, and dN/dS to obtain a general evolutionary pattern of selective pressure along the closely related lineages with gene loss. The mean dN/dS ratios were estimated by excluding genes with an extremely small estimation of dN or dS (< 0.001, which would always result in a very large dN/dS) [111]. The *P*-value was calculated as described above.

### Supplementary Information

**Additional file 1: Tables S1–S8. Table S1**: Collection, assembly, and accession numbers information of 93 Poales species. **Table S2**: Features of 93 Poales species plastomes. **Table S3**: Primes used for validation of four plastomes (*Anarthria humilis, Isolepis setacea, Xyris capensis* and *X.* *capensis* var. *schoenoides*) and sequence identity information of Sanger sequencing. **Table S4**: The permutation of number coded LCBs, the inversion numbers and inversion regions for each plastome. **Table S5:** Corresponding nucleotide models using the baseml of PAML for the 80PG-half matrix. **Table S6**: The positive selection test based on branch-site model. **Table S7**: Selective pressure analysis based on the free ratio model on the genes lost in certain lineages. **Table S8:** Collection information, sequencing information of plastid data for all taxa sampled used in phylogenetic tree.

**Additional file 2: Figs. S1–S9. Fig. S1.** The plastomic configuration of Poales. Numbers represent possible configurations based on the results of assembly software GetOrganelle. **Fig. S2.** Plastome gene maps of Poales. Genes on the inside and outside of the map are transcribed clockwise and transcribed counterclockwise, respectively. Different color genes represent different function. **Fig. S3.** Variation of GC content in each family of Poales. The graph displays the trends in GC content, wherein the purple dots indicate the mean value and the black lines connecting them represent GC content variation. **Fig. S4.** Verification of four species (*Anarthria humilis, Isolepis setacea, Xyris capensis* and *X.* *capensis* var. *schoenoides* plastomes) by PCR and corresponding readmapping graphs. The IR regions are indicated by black squares and the direction is indicated by a red arrow. The corresponding sequences of validation are denoted with blue border and the names are listed in blue text. **Fig. S5.** Heatmap of gene and intron content of 93 plastomes in Poales. Different colors represent gene deletions, gene copy number, the number of intron deletions and pseudogenes. The color box on the left represents the in formation of each family and clade of Poales, and the species with red text are incomplete plastomes. The yellow color bar at the bottom of the heatmap represents that these genes are all present in all plastomes of Poales, which have no mutation. **Fig. S6.** Scatter plot of repeat length for each family of Poales. **Fig. S7.** The Poales phylogram is based on 80PG-half matrix. **Fig. S8.** Correlation analysis of repeat number and plastomic size. The shaded area indicates 95% confidence intervals. **Fig. S9. A** Correlation analysis of repeat number and inversion number. **B** Correlation analysis of inversion number and arrangement distance. The shaded area indicates 95% confidence intervals.

**Additional file 3:** 80PG-half matrix.

**Additional file 4: **63 single-gene matrices used in select pressure analysis.

## Data Availability

All data generated or analyzed during this study are included in this published article, its supplementary information files, and publicly available repositories. The 60 assembled plastome sequences in our analyses were all submitted to the NCBI database, and accession numbers are listed in Additional file 1: Table S1.

## Abbreviations

AICc: Corrected Akaike information criterion
CAM: Crassulacean acid metabolism
DR: Direct repeat
sIR: Short inverted repeat
IR: Inverted repeat
LSC: Large single copy
SSC: Small single copy
RDR: Recombination-dependent replication
rDNA: Ribosomal DNA
tRNA: Genes and transfer RNA
