# Supplementary figures and images for: Unprecedented variation pattern of plastid genomes and the potential role in adaptive evolution in Poales
Source: BMC Biol. 2024 Apr 29;22:97. doi: 10.1186/s12915-024-01890-5 (PMC11057118; doi:10.1186/s12915-024-01890-5)

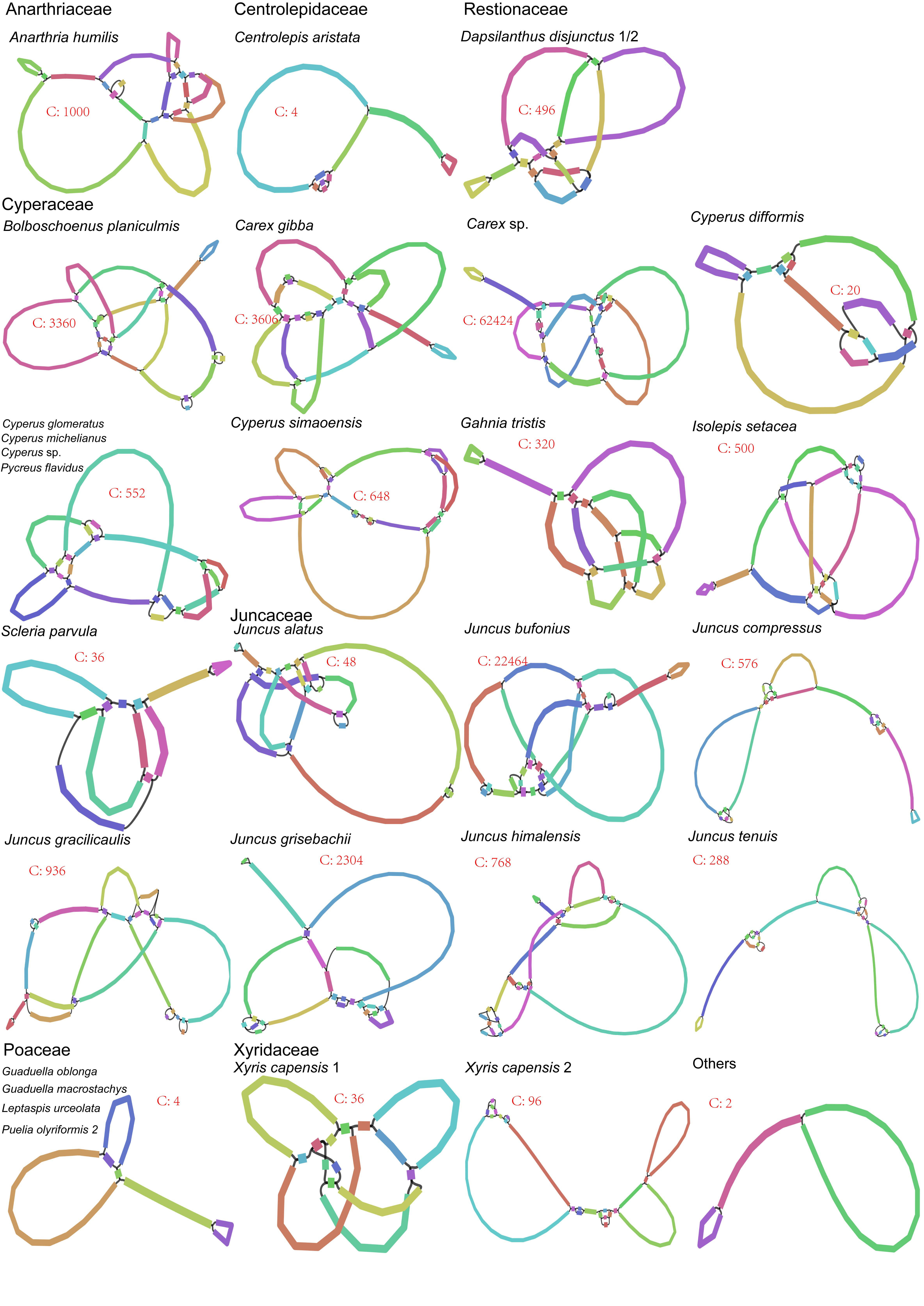

Supplement: Supplementary file 2 — Additional file 2: Figs. S1–S9. Fig. S1. The plastomic configuration of Poales. Numbers represent possible configurations based on the results of assembly software GetOrganelle. Fig. S2. Plastome gene maps of Poales. Genes on the inside and outside of the map are transcribed clockwise and transcribed counterclockwise, respectively. Different color genes represent different function. Fig. S3. Variation of GC content in each family of Poales. The graph displays the trends in GC content, wherein the purple dots indicate the mean value and the black lines connecting them represent GC content variation. Fig. S4. Verification of four species (Anarthria humilis, Isolepis setacea, Xyris capensis and X. capensis var. schoenoides plastomes) by PCR and corresponding readmapping graphs. The IR regions are indicated by black squares and the direction is indicated by a red arrow. The corresponding sequences of validation are denoted with blue border and the names are listed in blue text. Fig. S5. Heatmap of gene and intron content of 93 plastomes in Poales. Different colors represent gene deletions, gene copy number, the number of intron deletions and pseudogenes. The color box on the left represents the in formation of each family and clade of Poales, and the species with red text are incomplete plastomes. The yellow color bar at the bottom of the heatmap represents that these genes are all present in all plastomes of Poales, which have no mutation. Fig. S6. Scatter plot of repeat length for each family of Poales. Fig. S7. The Poales phylogram is based on 80PG-half matrix. Fig. S8. Correlation analysis of repeat number and plastomic size. The shaded area indicates 95% confidence intervals. Fig. S9. A Correlation analysis of repeat number and inversion number. B Correlation analysis of inversion number and arrangement distance. The shaded area indicates 95% confidence intervals. [file 12915_2024_1890_MOESM2_ESM.zip › Fig. S1-conformation of plastomes.jpg]

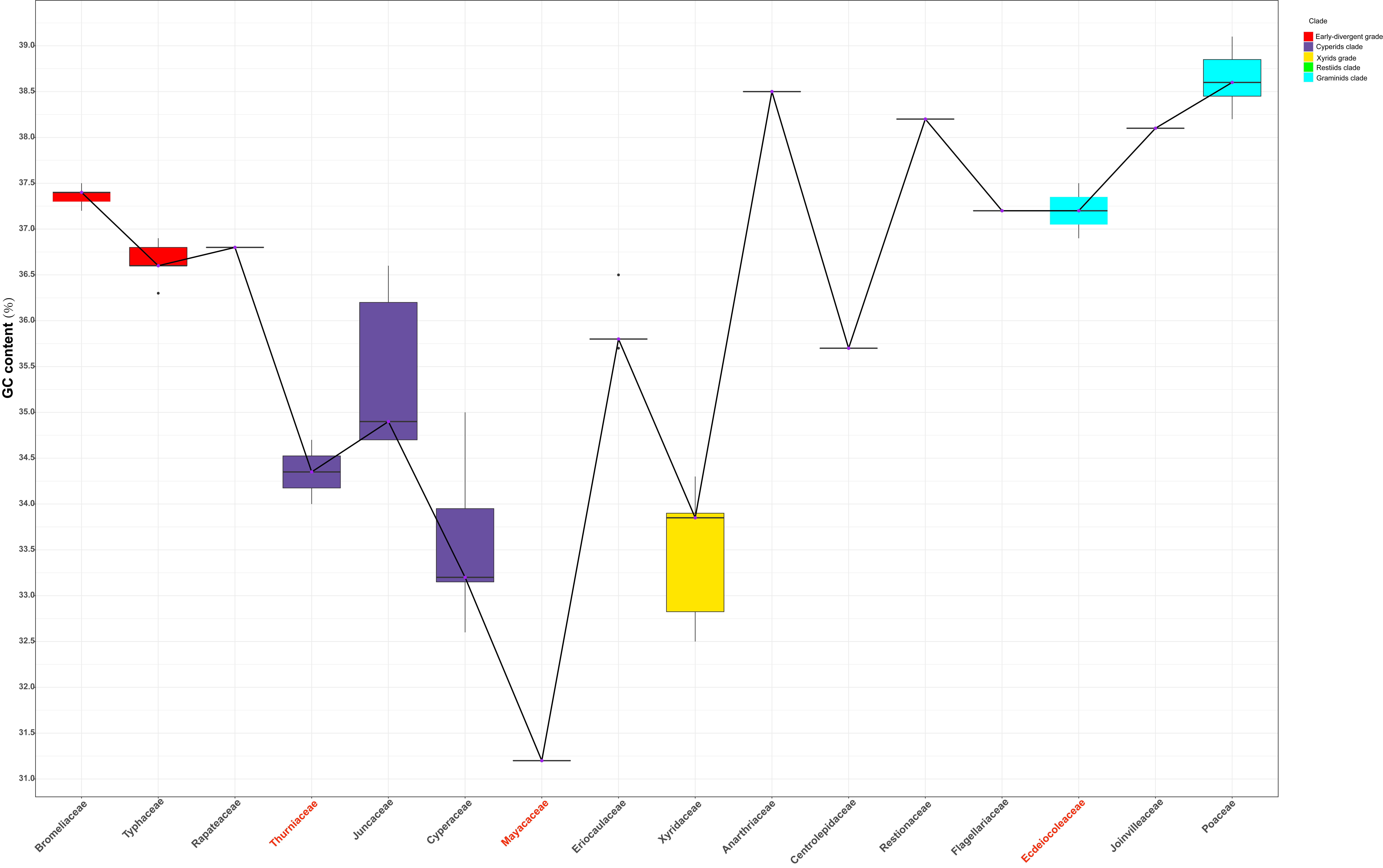

Supplement: Supplementary file 2 — Additional file 2: Figs. S1–S9. Fig. S1. The plastomic configuration of Poales. Numbers represent possible configurations based on the results of assembly software GetOrganelle. Fig. S2. Plastome gene maps of Poales. Genes on the inside and outside of the map are transcribed clockwise and transcribed counterclockwise, respectively. Different color genes represent different function. Fig. S3. Variation of GC content in each family of Poales. The graph displays the trends in GC content, wherein the purple dots indicate the mean value and the black lines connecting them represent GC content variation. Fig. S4. Verification of four species (Anarthria humilis, Isolepis setacea, Xyris capensis and X. capensis var. schoenoides plastomes) by PCR and corresponding readmapping graphs. The IR regions are indicated by black squares and the direction is indicated by a red arrow. The corresponding sequences of validation are denoted with blue border and the names are listed in blue text. Fig. S5. Heatmap of gene and intron content of 93 plastomes in Poales. Different colors represent gene deletions, gene copy number, the number of intron deletions and pseudogenes. The color box on the left represents the in formation of each family and clade of Poales, and the species with red text are incomplete plastomes. The yellow color bar at the bottom of the heatmap represents that these genes are all present in all plastomes of Poales, which have no mutation. Fig. S6. Scatter plot of repeat length for each family of Poales. Fig. S7. The Poales phylogram is based on 80PG-half matrix. Fig. S8. Correlation analysis of repeat number and plastomic size. The shaded area indicates 95% confidence intervals. Fig. S9. A Correlation analysis of repeat number and inversion number. B Correlation analysis of inversion number and arrangement distance. The shaded area indicates 95% confidence intervals. [file 12915_2024_1890_MOESM2_ESM.zip › Fig. S3-GC-content-line-1106.jpg]

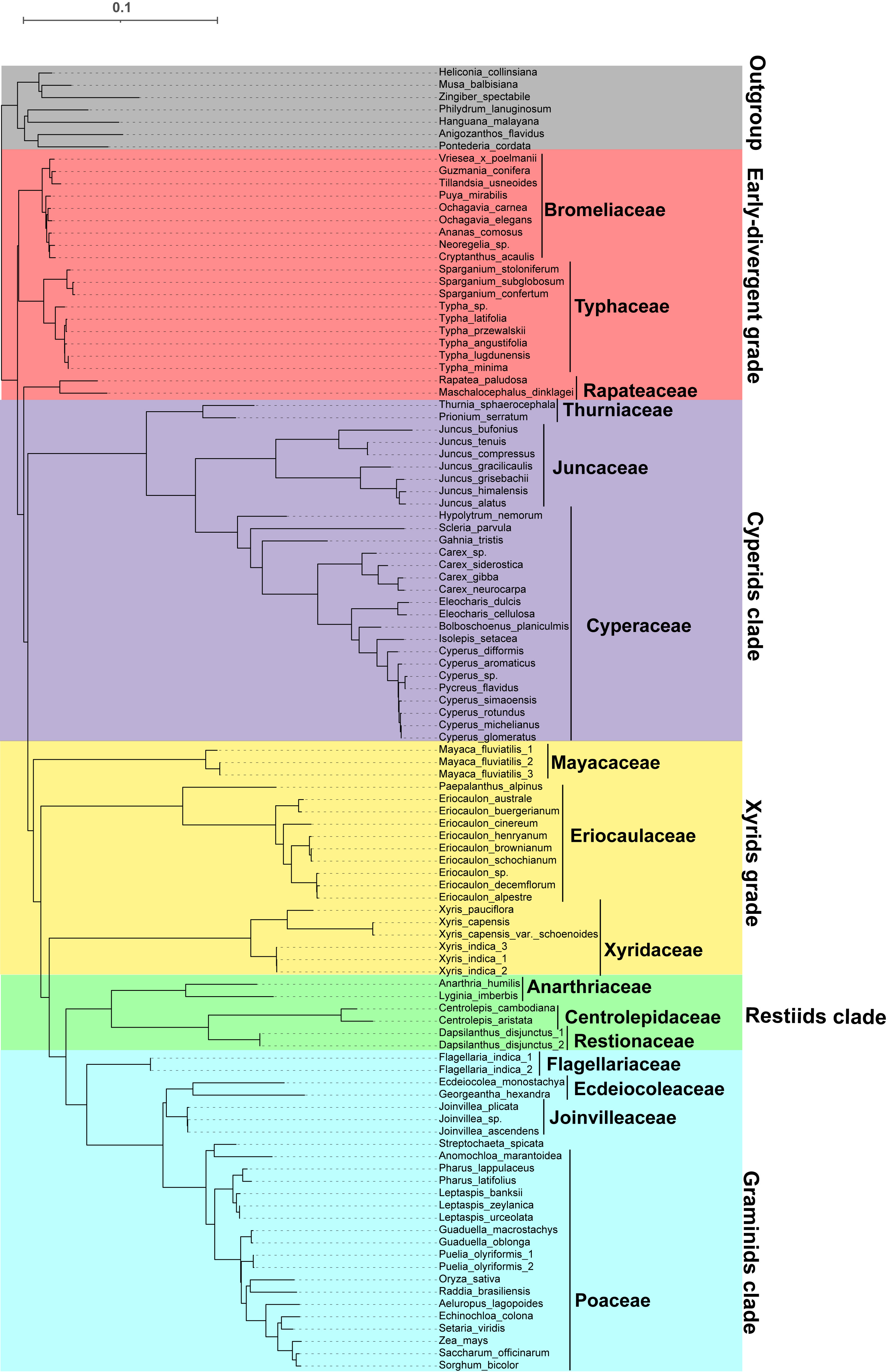

Supplement: Supplementary file 2 — Additional file 2: Figs. S1–S9. Fig. S1. The plastomic configuration of Poales. Numbers represent possible configurations based on the results of assembly software GetOrganelle. Fig. S2. Plastome gene maps of Poales. Genes on the inside and outside of the map are transcribed clockwise and transcribed counterclockwise, respectively. Different color genes represent different function. Fig. S3. Variation of GC content in each family of Poales. The graph displays the trends in GC content, wherein the purple dots indicate the mean value and the black lines connecting them represent GC content variation. Fig. S4. Verification of four species (Anarthria humilis, Isolepis setacea, Xyris capensis and X. capensis var. schoenoides plastomes) by PCR and corresponding readmapping graphs. The IR regions are indicated by black squares and the direction is indicated by a red arrow. The corresponding sequences of validation are denoted with blue border and the names are listed in blue text. Fig. S5. Heatmap of gene and intron content of 93 plastomes in Poales. Different colors represent gene deletions, gene copy number, the number of intron deletions and pseudogenes. The color box on the left represents the in formation of each family and clade of Poales, and the species with red text are incomplete plastomes. The yellow color bar at the bottom of the heatmap represents that these genes are all present in all plastomes of Poales, which have no mutation. Fig. S6. Scatter plot of repeat length for each family of Poales. Fig. S7. The Poales phylogram is based on 80PG-half matrix. Fig. S8. Correlation analysis of repeat number and plastomic size. The shaded area indicates 95% confidence intervals. Fig. S9. A Correlation analysis of repeat number and inversion number. B Correlation analysis of inversion number and arrangement distance. The shaded area indicates 95% confidence intervals. [file 12915_2024_1890_MOESM2_ESM.zip › Fig. S7-Poales phylogram.jpg]

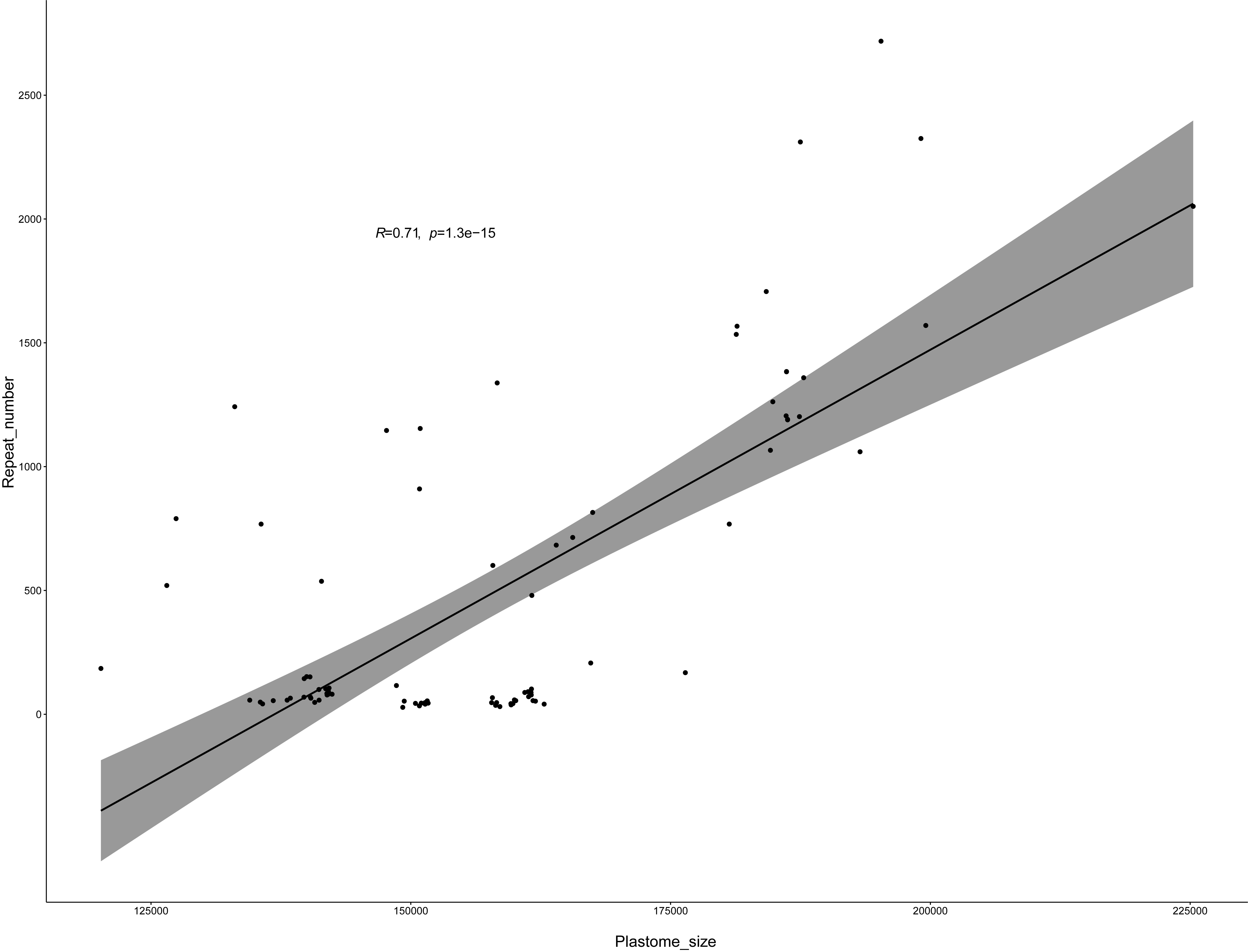

Supplement: Supplementary file 2 — Additional file 2: Figs. S1–S9. Fig. S1. The plastomic configuration of Poales. Numbers represent possible configurations based on the results of assembly software GetOrganelle. Fig. S2. Plastome gene maps of Poales. Genes on the inside and outside of the map are transcribed clockwise and transcribed counterclockwise, respectively. Different color genes represent different function. Fig. S3. Variation of GC content in each family of Poales. The graph displays the trends in GC content, wherein the purple dots indicate the mean value and the black lines connecting them represent GC content variation. Fig. S4. Verification of four species (Anarthria humilis, Isolepis setacea, Xyris capensis and X. capensis var. schoenoides plastomes) by PCR and corresponding readmapping graphs. The IR regions are indicated by black squares and the direction is indicated by a red arrow. The corresponding sequences of validation are denoted with blue border and the names are listed in blue text. Fig. S5. Heatmap of gene and intron content of 93 plastomes in Poales. Different colors represent gene deletions, gene copy number, the number of intron deletions and pseudogenes. The color box on the left represents the in formation of each family and clade of Poales, and the species with red text are incomplete plastomes. The yellow color bar at the bottom of the heatmap represents that these genes are all present in all plastomes of Poales, which have no mutation. Fig. S6. Scatter plot of repeat length for each family of Poales. Fig. S7. The Poales phylogram is based on 80PG-half matrix. Fig. S8. Correlation analysis of repeat number and plastomic size. The shaded area indicates 95% confidence intervals. Fig. S9. A Correlation analysis of repeat number and inversion number. B Correlation analysis of inversion number and arrangement distance. The shaded area indicates 95% confidence intervals. [file 12915_2024_1890_MOESM2_ESM.zip › Fig. S8 correlation-repeat_size_1018.jpg]

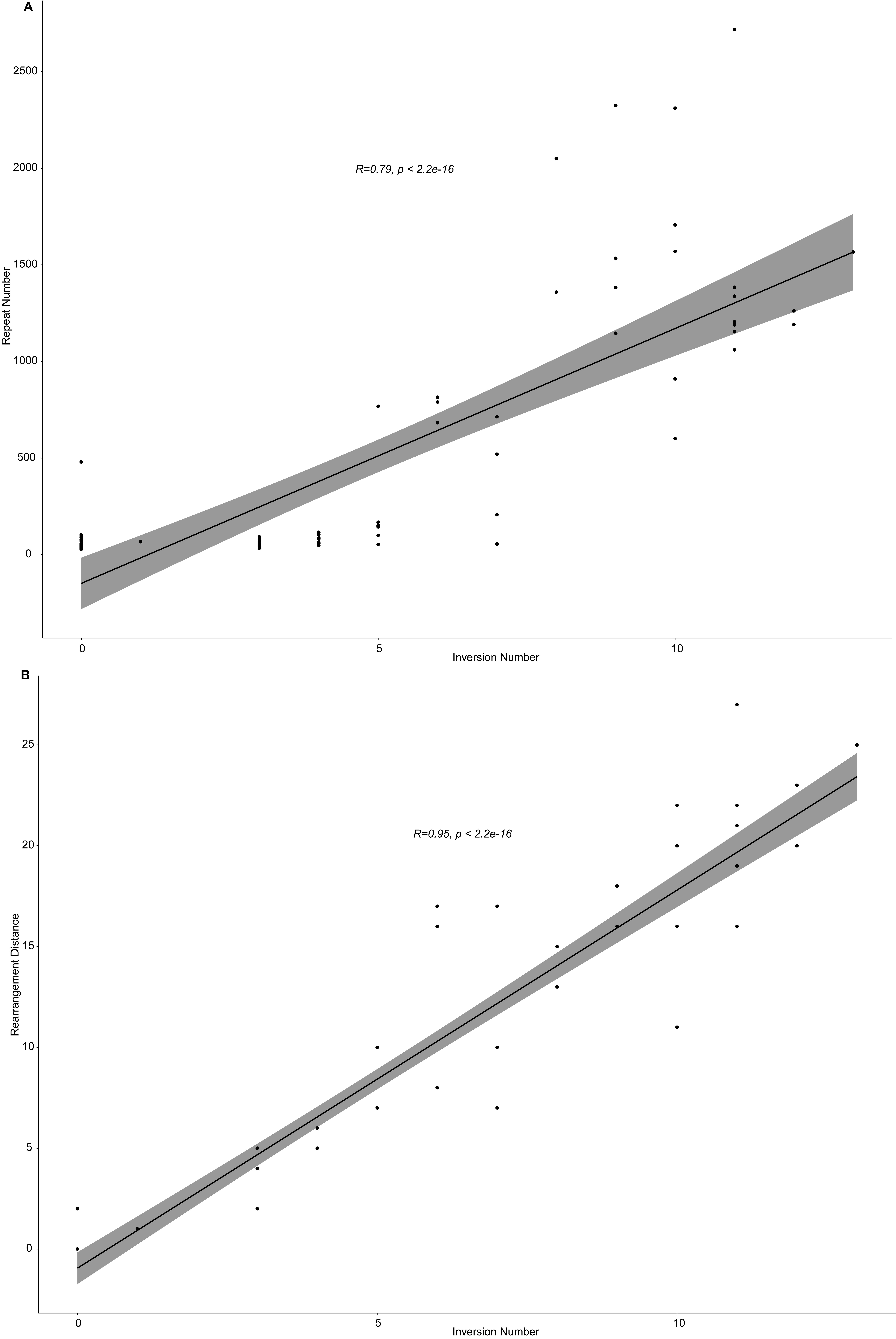

Supplement: Supplementary file 2 — Additional file 2: Figs. S1–S9. Fig. S1. The plastomic configuration of Poales. Numbers represent possible configurations based on the results of assembly software GetOrganelle. Fig. S2. Plastome gene maps of Poales. Genes on the inside and outside of the map are transcribed clockwise and transcribed counterclockwise, respectively. Different color genes represent different function. Fig. S3. Variation of GC content in each family of Poales. The graph displays the trends in GC content, wherein the purple dots indicate the mean value and the black lines connecting them represent GC content variation. Fig. S4. Verification of four species (Anarthria humilis, Isolepis setacea, Xyris capensis and X. capensis var. schoenoides plastomes) by PCR and corresponding readmapping graphs. The IR regions are indicated by black squares and the direction is indicated by a red arrow. The corresponding sequences of validation are denoted with blue border and the names are listed in blue text. Fig. S5. Heatmap of gene and intron content of 93 plastomes in Poales. Different colors represent gene deletions, gene copy number, the number of intron deletions and pseudogenes. The color box on the left represents the in formation of each family and clade of Poales, and the species with red text are incomplete plastomes. The yellow color bar at the bottom of the heatmap represents that these genes are all present in all plastomes of Poales, which have no mutation. Fig. S6. Scatter plot of repeat length for each family of Poales. Fig. S7. The Poales phylogram is based on 80PG-half matrix. Fig. S8. Correlation analysis of repeat number and plastomic size. The shaded area indicates 95% confidence intervals. Fig. S9. A Correlation analysis of repeat number and inversion number. B Correlation analysis of inversion number and arrangement distance. The shaded area indicates 95% confidence intervals. [file 12915_2024_1890_MOESM2_ESM.zip › Fig. S9-correlation of R-I-A.jpg]
